# Silver Binding Nucleolar Organizer Regions Dots in Oral Leukoplakia with Epithelial Dysplasia and Oral Squamous Cell Carcinoma: An *In Vivo* Study

**DOI:** 10.1155/2014/479187

**Published:** 2014-04-30

**Authors:** Fahad Mansoor Samadi, Bastain Thattil Sebastian, Anil Singh, Shaleen Chandra, Shadab Mohammad, Arun Singh, Thippeswamy Halappa, Firoza Samadi

**Affiliations:** ^1^Department of Oral Pathology & Microbiology, King George's Medical University, Lucknow, India; ^2^Department of Oral Pathology & Microbiology, Mahe Institute of Dental Sciences, Palloor, Mahe, Puducherry, India; ^3^Department of Oral Pathology & Microbiology, Saraswati Dental College, Lucknow, India; ^4^Department of Oral & Maxillofacial Surgery, King George's Medical University, Lucknow, India; ^5^Department of Oral Pathology & Microbiology, Kothiwal Dental College & Research Centre, Moradabad, Uttar Pradesh, India; ^6^Department of Oral Pathology & Microbiology, Babu Banarsi Das College of Dental Sciences, Lucknow, India; ^7^Department of Pedodontics, Career Post Graduate Institute of Dental Sciences and Hospital, Lucknow, Uttar Pradesh, India

## Abstract

Silver binding nucleolar organizer regions (AgNOR) in normal oral mucosa (NOM), oral leukoplakia with epithelial dysplasia (ED), and oral squamous cell carcinoma (OSCC) were studied. The mean AgNOR count per nucleus increased from NOM to ED to OSCC. Tissue showing ED in oral leukoplakia and OSCC cases showed higher counts, wider scatter, and smaller size of AgNOR dots in the nuclei. The study seems to suggest that time method has some potential in distinguishing between NOM and oral leukoplakia with ED and OSCC. Studies of larger numbers are needed to arrive at more substantial conclusions.

## 1. Introduction

Leukoplakias are group of lesions of different etiologies and most common potentially malignant oral lesions. Epithelial dysplasias (ED) are malignant changes, seen in leukoplakias [1]. Oral squamous cell carcinoma (OSCC) is a malignant neoplasm arising from mucosal epithelium. Its early detection is crucial. To date, no clear markers have evolved to distinguish them [2]. Patterns of nucleolar organizer regions (NOR) as visualized by silver staining (AgNOR) vary with different cell cycle stages. These are replicatory markers useful in diagnosing oral leukoplakia [3].

The purpose of this study was to quantify AgNOR count in tissue sections of normal oral mucosa (NOM), ED, and OSCC.

## 2. Materials and Method

The study consisted of three groups divided into Group I (control group, ten cases of NOM), Group II (30 cases of ED—ten each of mild, moderate, and severe cases), and Group III (30 cases of OSCC—ten each of well differentiated, moderately differentiated, and poorly differentiated).

Two histological sections of five *μ*m thickness were obtained from each case. One section was stained with hematoxylin and eosin (H&E) stain and the other with silver nitrate solution. AgNOR staining was done according to modified procedure of Smith and Crocker [4]. After the histological grading of the lesions with H&E sections, corresponding silver stained sections were subjected to quantitative analysis of AgNOR dots and carried out under X100 oil immersion lens.

In each section, five different fields with the most representative areas were selected, which included five largest nuclei with clear outline in each field.

The numbers of silver stained, black dots were counted in each nucleus. AgNOR dots seen in a clump were counted as single AgNOR dot. Further mean AgNOR count in each case was determined and tabulated and subjected to statistical analysis.

## 3. Results

Table 1 shows the mean AgNOR count in different study groups.

Table 2 shows the analysis of variance of mean AgNOR count in different study groups.

Table 3 shows the intergroup comparison between the different study groups.

Intergroup comparison revealed statistically significant difference between different study groups specifying that it was possible to differentiate different diagnostic groups with the help of mean AgNOR count. The order of mean AgNOR count in relation to the diagnostic groups was as follows.

NOM < mild ED < moderate ED < severe ED < well differentiated OSCC < moderately differentiated OSCC < poorly differentiated OSCC.

## 4. Discussion

Leukoplakia is a predominantly white lesion of the oral mucosa that cannot be characterized as any other definable lesion [5]. It is a clinical term and it is recommended that a histological report should always include a statement on the presence or absence of ED, along with assessment of its severity [6]. ED is more or less correlating with a clinical nonhomogeneous erythroleukoplakic subtype, which seems to be the most important indicator of malignant potential [7].

OSCC is a malignant neoplasm of oral mucosal epithelium. Reference [8] is the sixth most common malignancy and a major cause of cancer morbidity and mortality worldwide [9]. It is recognized as a disease resulting from genetic damage, leading to uncontrolled cell proliferation of damaged cells [8]. Oral cancer patients share the common high risk of developing multiple or sequential primary carcinomas within the upper aerodigestive tract due to field cancerization. Early detection of premalignant and/or neoplastic lesions is therefore crucial for improving the long-term prospective of patients suffering from OSCC. To date, no clear markers for distinguishing mild, moderate, and severe ED have evolved and histological criteria for diagnosing a “dysplastic” lesion are still subjective [2].

NOR are loops of DNA on the short arms of acrocentric chromosomes that presumably are associated with ribosomal RNA activity, protein synthesis, and cell proliferations. The patterns of neither NOR by size and distribution, as visualized by silver staining (AgNOR), are known to vary with different cell cycle stages. The number of AgNOR increases from the early G_1 _phase to late S/G_2_ phases. The number of AgNOR at any given stage in the cell cycle appears to be inversely proportional to the cell cycle time, that is, the higher the amount of AgNOR, the shorter the cell cycle time [3]. AgNOR have been shown to be replicatory markers. Mean AgNOR count can be useful in diagnosing histological features representing oral leukoplakia and has been shown to correlate with clinical outcomes of various cancers [10]. An obvious feature of all oral cancers is excessive proliferation of oral keratinocytes. Initially keratinocyte proliferation may be confined to the epithelial compartment resulting in a thickened and disorganized epithelium. Individual keratinocytes show nuclear hyperchromatism (dark staining), nuclear pleomorphism (abnormal shape), enlarged nucleoli, increased nuclear: cytoplasmic ratio, increased mitoses, atypical mitoses, and multinucleation [11]. Even though H&E staining is routinely used for diagnosing carcinoma histologically, in some instances, histopathological features are not defined sufficiently to determine the true nature of these disturbances. Thus, alternative methods like silver colloid technique for staining NOR in nucleus are a helpful histochemical technique to provide more information about the cellular status [12].

Several studies have demonstrated that AgNOR being replicatory markers may be useful in diagnosis of various neoplasms. OSCC can be distinguished from normal epithelium by AgNOR count. Furthermore, some studies observed that it might be possible to use AgNOR to distinguish mild and moderate ED. AgNOR technique, being inexpensive and its results easily reproducible, can be very effective in resource-poor setting [2].

NORs were first detected on Giemsa banding as “achromatic gaps” with much reduced staining. The areas are not, of course, genuine gaps but are areas of specialized chromosomal configuration. The major advance in the demonstration of NOR came with the discovery that they are highly argyrophilic, because of the properties of some of their associated proteins. Accordingly, one of the most popular methods for demonstrating these areas has made use of the binding of Ag+ ions and the structures revealed have been assigned the rather unattractive name AgNOR. It was shown, then, that the achromatic gap areas on the acrocentric chromosomes were argyrophilic and the silver binding method became a standard. It was found repeatedly that the numbers of histochemically stained NOR in interphase cells represented their proliferative state rather than any other variable, such as, for example, protein synthetic level [13].

The quantification of these has been a useful method in diagnostic pathology especially in the differential diagnosis between benign and malignant tumors and helpful in recognizing limitrophic lesions [12].

Our study was carried out to determine and compare mean AgNOR counts in normal oral mucosa and oral leukoplakia with ED and OSCC. Comparison was also done between different degrees of ED in oral leukoplakia group and different stages of OSCC.

Gomez suggested that AgNOR can be useful as a compliment to histopathological examination and to a certain point indicates degree of malignant/dysplastic alterations [14].

The results of the present study showed an increase in mean AgNOR count in both oral leukoplakia with ED and OSCC over NOM. AgNOR in OSCC were smaller, more numerous, and more scattered throughout the nuclei than in the normal epithelium. Leukoplakia with ED also showed smaller and more scattered dots. These results were in accordance with the study conducted by Chattopadhyay [15]. The higher AgNOR counts and smaller more widely scattered dots in dysplastic leukoplakia appear to be a significant finding. It suggests that as the lesion becomes more dysplastic and ultimately malignant, the AgNOR count tends to increase and AgNOR dot size decreases as the dots are more widely scattered over the nuclei. According to Chattopadhyay the AgNOR pattern suggests strong correlation of AgNOR character with cell replicatory capacity and cellular differentiation. The highly replicatory areas of basal cell hyperplasia have shown high count, high scatter, and smaller AgNOR, factors which reverse in the nonreplicatory higher layers where cellular differentiation becomes more evident [16].

**Figure 1:**
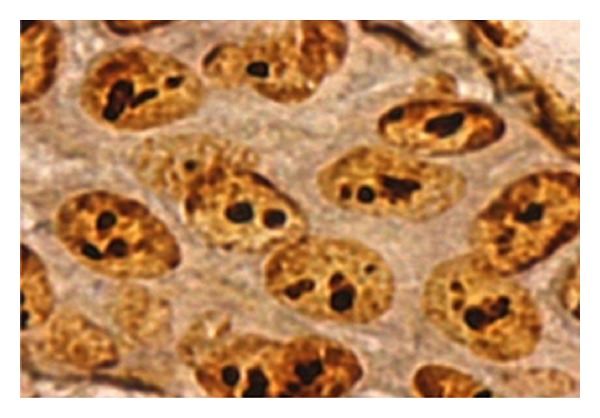
AgNOR dots seen in mild epithelial dysplasia under oil immersion lens (100x).

The results of this study revealed mean AgNOR counts in oral leukoplakia with ED to be higher (5.602 ± 0.456) than in the control group (2.572 ± 0.121) (Figure 1). It correlates with the findings of Chattopadhyay [15].

Ray et al. in their studies on premalignant lesions showed higher mean AgNOR counts in leukoplakia as compared to normal epithelium, [10] which was similar to our study which showed increase in mean AgNOR counts from mild to severe epithelial dysplasia in various leukoplakic lesions. Warnakulasuriya and Johnson also revealed more dispersed counts of AgNOR in all types of dysplasias. [17].

In the present study, out of ten cases of mild, moderate, and severe dysplasia, the mean AgNOR counts were 5.184, 5.628, and 5.993, respectively. This indicated that the higher the proliferative activity of cells, the greater the grade of dysplasia with increase in the AgNOR counts. In accordance with the present study, Xie et al. showed significant differences of mean AgNOR counts between normal epithelium and mild and severe dysplasia [3]. Study of Pandit and Aithal also revealed the same [18].

**Figure 2:**
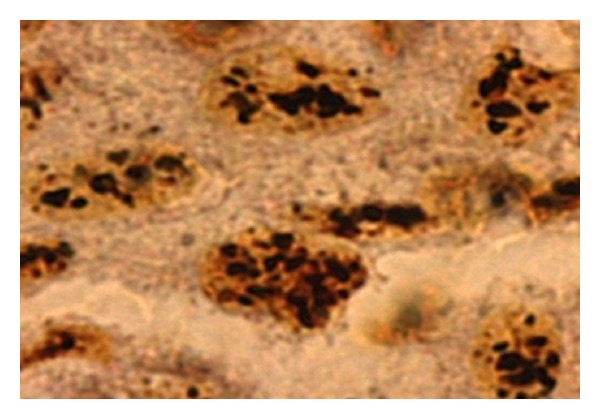
AgNOR dots seen in poorly differentiated oral squamous cell carcinoma under oil immersion lens (100x).

The findings of our study showed AgNOR counts to be much higher in OSCC, when compared to oral leukoplakia with ED (Figure 2). Similarly in our study there has been a progressive increase in the values of total AgNOR from mild to moderate to severe ED. Chattopadhyay et al. stated that AgNOR counts can be a useful parameter to determine the presence of dysplasia. With respect to mean AgNOR counts, leukoplakia occupies a distribution between that of normal epithelium and squamous cell carcinoma [19]. The AgNOR technique therefore seems to be a strong potential candidate for a test that could provide objective criteria for marking dysplasia. Kahn et al. also revealed that the average maximum AgNOR count shows a tendency to increase with each increasing grade of dysplasia [20]. Similar findings were reported by Cabrini et al. and Chattopadhyay who showed that the number of AgNOR per nucleus increased from normal oral mucosa to squamous cell carcinoma [15, 21].

## 5. Conclusion

Mean AgNOR counts allow discrimination between NOM, oral leukoplakia with ED, and well differentiated OSCC. Higher values in severe ED may suggest a greater risk of malignant transformation.

AgNOR counting had a significant correlation between normal and malignant epithelium and with stage of disease. High AgNOR expression has poorer prognostic indication than lower AgNOR values.

An association between cell proliferation and AgNOR counting in OSCC indicates that quantitative analysis of the mean number of NORs per nucleus represents a useful and inexpensive method for measuring cell proliferation in OSCC. Routine use of AgNOR stain in dysplastic lesions and early OSCC helpful for unequivocal diagnosis of these lesions which otherwise remained highly subjective in nature.

Further studies are warranted to quantify AgNOR probably in the invasive tumor front especially in the OSCC as the prognostic relevance of invasive tumor front has been established in classical histopathological studies. The findings of such studies could be of immense help in correlating the clinical course of the disease and may throw light into overall survival and disease-free survival over a period of time.

## Figures and Tables

**Table 1:** Mean AgNOR count and standard deviation in different study groups.

Study groups	Mean ± S.D.
NOM	2.572 ± 0.121
Mild ED	5.184 ± 0.095
Moderate ED	5.628 ± 0.477
Severe ED	5.993 ± 0.262
Well differentiated OSCC	7.351 ± 0.285
Moderately differentiated OSCC	8.060 ± 0.175
Poorly differentiated OSCC	8.810 ± 0.302

**Table 2:** Analysis of variance of mean AgNOR count in different study groups.

	Sum of squares	Df	Mean square	*F*
Between study groups	258.624	6	43.104	724.530
Within study groups	3.748	63	0.059	

Total	262.372	69		

Analysis of variance revealed a statistical significance in the mean AgNOR count among different groups (*P* < 0.001).

**Table 3:** Intergroup comparison.

Group	Mean ± S.D.	Statistical significance
Group I	2.572 ± 0.121	NOM versus ED: *t* = 20.605; *P* < 0.001
Group III	5.602 ± 0.456	NOM versus OSCC: *t* = 26.185; *P* < 0.001
Group III	8.073 ± 0.655	ED versus OSCC: *t* = 16.958; *P* < 0.001
